# Paradoxical and bimodal immune‐mediated dermatological side effects of TNF‐α inhibitors: A comprehensive review

**DOI:** 10.1111/srt.13718

**Published:** 2024-05-03

**Authors:** Elham Behrangi, Farzan Moodi, Alireza Jafarzadeh, Azadeh Goodarzi

**Affiliations:** ^1^ Department of Dermatology Rasool Akram Medical Complex Clinical Research Development Center (RCRDC) School of Medicine Iran University of Medical Sciences (IUMS) Tehran Iran; ^2^ Skin and Stem Cell Research Center Tehran University of Medical Sciences Tehran Iran; ^3^ School of Medicine Iran University of Medical Sciences Tehran Iran

**Keywords:** eczema, eosinophilic cellulitis, hidradenitis supparativa, paradoxical side effects, psoriasis, sarcoidosis, TNF alpha inhibitors

## Abstract

**Introduction:**

Due to the increasing prevalence of immune‐mediated diseases such as psoriasis, lichen planus, rheumatoid arthritis and inflammatory bowel disease, dermatologists have turned to new biologic drugs known as DMARDs (disease‐modifying anti‐rheumatic drugs) in recent years.

**Areas Covered:**

In this study, we evaluate the immune‐mediated dermatological side effects of DMARDS by reviewing and analyzing previous peer‐reviewed research on the effects of TNF‐α inhibitors in the treatment of skin diseases, as well as adverse effects of these drugs and some of the main causes of these effects.

**Expert Opinion:**

DMARDs are very effective in improving control of the above diseases. TNF‐α inhibitors are an important group of DMARDs that are widely used. The paradoxical adverse events (PAEs) associated with the use of TNF‐α inhibitors are divided into three categories: true paradoxical, borderline paradoxical, and non‐paradoxical. True PAEs include conditions for which TNF‐α inhibitors are approved for treatment. Borderline PAEs are considered to occur with this class of drugs for which there is no definite approval but for which there is sufficient evidence. Although these events are rare, early recognition of the accused drug and appropriate decision‐making may prevent progression of complications and irreversible side effects.

**Abbreviation**
DMARD: disease‐modifying anti‐rheumatic drug
IBD: inflammatory bowel disease
PAE: paradoxical adverse event
TNF‐α: tumor necrosis factor alpha

## 1 INTRODUCTION

With the increase in the incidence and prevalence of immune‐mediated diseases, clinicians tend to use new biologic agents, known as DMARDs, to control and treat these diseases with minimal side effects. These immune‐mediated diseases include psoriasis, lichen planus, rheumatoid arthritis and inflammatory bowel disease (IBD). An important group of DMARDs commonly used by clinicians are TNF‐α inhibitors.^1^ In the 1960s, scientists discovered the presence of TNF‐α as a factor with necrotizing activity in tumor cells in mice^2^; however, it was not fully discovered until 1984 by Aggarwal and colleagues when they evaluated cytokines secreted by macrophages and T lymphocytes.^3^ In 1985, Maini and Feldmann demonstrated the role of TNF‐α in rheumatoid arthritis by examining the cytokines secreted in this disease. These findings formed the basis for the first clinical trial in patients with rheumatoid arthritis using the monoclonal antibody cA2, now known as infliximab.^2^ The mechanism of action of TNF‐α inhibitors is to prevent TNF‐α types 1 and 2 from binding to their receptors. Additionally, this class of drugs can reduce the expression of high mobility group box 1 (HMGB1), thereby reducing inflammation.^2^, ^4^, ^5^ In some diseases, such as psoriasis and rheumatoid arthritis, these drugs are approved by the FDA; in others, such as pyoderma gangrenosum and juvenile idiopathic arthritis, they are used off‐label.^4^ Gradually, it became clear that TNF‐α inhibitors can paradoxically cause the same diseases they treat.^6^ The definition and classification of the mentioned complications are shown in Table 1.

**TABLE 1:** Classification of dermatological side effects of TNF‐α inhibitors.

Examples	Definition	Classification
Psoriasis, hidradenitis suppurativa (4)	Complications that occur during the treatment of another disease, while TNF‐α inhibitors drugs are themselves the approved treatment for this complication. In addition, exacerbations of cases treated with this drug category are also included in this definition^9^, ^10^	True paradoxical adverse effects
Sarcoidosis^9^	Complications that should respond to this drug class based on their pathophysiology, but for which the FDA has not yet granted treatment authorization^9^	Borderline or possible paradoxical adverse effects
Eczema, Eosinophilic cellulitis^9^	This category of side effects includes those caused by taking these medicines and TNF‐α inhibitors are not a logical treatment for these side effects^9^	Non‐paradoxical adverse effects

In 2004, the first case of psoriasis was reported after the use of TNF‐α inhibitors.^6^ Over time, other reports of PAEs were published that were not limited to the skin but were related to organs outside the skin, including a case of IBD after treatment for ankylosing spondylitis reported in 2007.^7^ About 25% of adverse reactions were dermatological reactions such as alopecia, erythema nodosum, immune bullous disorders, lichen planus, lichenoid drug reactions, erythema multiforme, Stevens Johnson, toxic epidermolysis necrolysis, vitiligo, neutrophilic dermatosis, and eosinophilic dermatosis.^8^ This shows the great importance of dermatological events caused by TNF‐α inhibitors. Our initial search revealed that the published review articles were not comprehensive enough and their last publication date was long ago. Given the increasing use of TNF‐α inhibitors, physicians, especially dermatologists, should become more familiar with these adverse events, and the present study was conducted for this purpose. Explanations of the mentioned complications are listed in Table 2.

**TABLE 2:** Dermatological reactions caused by TNF‐α inhibitors.

Treatment	Risk factors	The most common manifestation of the disease	Accused drug	Time interval between occurrence of lesion and start of medication	Prevalence	Disease
Mild: topical steroid ± vitamin D analog or keratolytic agent Severe: discontinue or change drug ± topical therapy/cyclosporine/methotrexate/phototherapy	Female gender Smoking	Palmoplantar pustular lesions	Adalimumab Certolizumab Infliximab	6–12 Months	The occurrence of a new lesion: common Exacerbation of previous lesion: rare	Psoriasis^11^, ^12^, ^13^, ^14^
Hurley 1: Start of specific treatment + continuation of medication Hurley 2/3: Discontinuation of medication	–	–	Adalimumab	12 Months	Rare	Hidradenitis suppurativa^15^, ^16^
Topical treatment ± discontinuation of drug ± Ustekinumab/ Tofacitinib	–	Alopecia areata, Psoriatic alopecia	Adalimumab Etanercept	11 Months	Rare	Alopecia^17^, ^18^
Discontinue drug if systemic involvement + topical/systemic steroid ± immunosuppressant	Female gender	Skin rashes	Infliximab	10 Months	Rare	Lupus^19^, ^20^, ^21^, ^22^
Discontinuation of drug + systemic steroid ±‐ methotrexate	–	Skin rashes and muscle weakness	Adalimumab Etanercept Infliximab	2 Months– 4 years	Very rare	Dermato‐myositis^23^, ^24^, ^25^, ^26^
Topical steroid ± calcineurin inhibitor	History of atopy	Non‐specific	Infliximab	7 Months	Common	Eczema^27^, ^28^, ^29^
Drug discontinuation + low‐dose systemic steroid/topical steroid or dapsone	–	Indurated plaque around the injection site	Adalimumab	A few hours to a week	Rare	Wells syndrome^30^, ^31^, ^32^, ^33^, ^34^, ^35^
Discontinuation of drug + initiation of specific treatment	–	Blisters on the trunk and limbs	Adalimumab Etanercept Infliximab	2 weeks–3 years	Very rare	Autoimmune bullous diseases^34^, ^35^, ^36^, ^37^, ^38^, ^39^, ^40^, ^41^, ^42^, ^43^, ^44^
Discontinuation of drug + topical/systemic steroid ± immunosuppressant	–	Generalized skin involvement with limbs preference	Adalimumab Etanercept Infliximab Certolizumab	3 weeks–24 months	Rare	Lichen planus and lichenoid drug reactions^45^, ^46^, ^47^, ^48^, ^49^, ^50^
Discontinuation of drug + topical or systemic steroid depending on severity, methylprednisolone or biologic drug in severe cases	–	Similar to the typical form but multiple with generalized involvement	All of TNF‐α inhibitors	3–6 Months	Very rare	Neutrophilic dermatoses^51^, ^52^, ^53^, ^54^, ^55^, ^56^, ^57^, ^58^, ^59^, ^60^
Discontinuation of medication + initiation of specific treatment	–	Erythema multiforme	Adalimumab Etanercept Infliximab	few days–8 weeks	Very rare	Erythema multiform, Stevens‐Johnson, toxic epidermal necrolysis (TEN).^61^, ^62^, ^63^, ^64^, ^65^, ^66^
Discontinuation of the drug + initiation of systemic corticosteroids	–	Cutaneous small vessels vasculitis	Etanercept Infliximab	9.5 Months	Rare	Vasculitis ^67^, ^68^, ^69^, ^70^, ^71^, ^72^
Mild: topical steroid ± vitamin D analog or keratolytic agent Severe: discontinue or change drug ± topical therapy/cyclosporine/methotrexate/phototherapy	Female gender Smoking	Palmoplantar pustular lesions	Adalimumab Certolizumab Infliximab	6–12 Months	The occurrence of a new lesion: common Exacerbation of previous lesion: rare	Psoriasis^11^, ^12^, ^13^, ^14^
Hurley 1: Start of specific treatment + continuation of medication Hurley 2/3: Discontinuation of medication	–	–	Adalimumab	12 Months	Rare	Hidradenitis suppurativa^15^, ^16^
Topical treatment ± discontinuation of drug ± Ustekinumab/ Tofacitinib	–	Alopecia areata, psoriatic alopecia	Adalimumab Etanercept	11 Months	Rare	Alopecia^17^, ^18^
Discontinue drug if systemic involvement + topical/systemic steroid ± immunosuppressant	Female gender	Skin rashes	Infliximab	10 Months	Rare	Lupus^19^, ^20^, ^21^, ^22^
Discontinuation of drug + systemic steroid ± methotrexate	–	Skin rashes and muscle weakness	Adalimumab Etanercept Infliximab	2 Months– 4 years	Very rare	Dermato‐myositis^23^, ^24^, ^25^, ^26^
Topical steroid ± calcineurin inhibitor	History of atopy	Non‐specific	Infliximab	7 Months	Common	Eczema^27^, ^28^, ^29^
Drug discontinuation + low‐dose systemic steroid/topical steroid or dapsone	–	Indurated plaque around the injection site	Adalimumab	A few hours to a week	Rare	Wells syndrome^30^, ^31^, ^32^, ^33^, ^34^, ^35^
Discontinuation of medication in cases of previous lesion + specific treatment	Less than 40 years old	Non‐segmental involvement, preferably trunk and thigh	Adalimumab Etanercept Infliximab	14 Months	Rare	Vitiligo^73^, ^74^, ^75^

**CAPSULE SUMMARY**

**What is known?**

In medicine, including dermatology, numerous therapeutic effects of TNF‐α inhibitors in the treatment of various diseases are known. The wide use of the mentioned drugs in the treatment of various diseases makes it necessary for physicians to know their possible side effects. Among these side effects, there are true paradoxical dermatological side effects, which means TNF‐α inhibitors that have been approved as a treatment option for the treatment of the complication that has developed. Another category of dermatological complications that can be expected with treatment with the TNF‐α inhibitors are borderline or possible paradoxical adverse effects, meaning that TNF‐α inhibitors may be suggested as a treatment option for the complication that has occurred but has not been approved by the FDA for its treatment. The third group includes skin complications that cannot be treated with the above drugs due to pathophysiology and are referred to as non‐paradoxical adverse effects. Examples of most common side effects mentioned in the above three groups are Psoriasis, sarcoidosis and Eczema, respectively.

**What does this study add?**

In this study, the dermatological side effects of TNF‐α inhibitors were discussed in detail. Reviewing numerous and reliable articles that focus more on paradoxical side effects, the prevalence of each of the above side effects, the timing of the appearance of lesions after the use of the drug, and the most common manifestations of skin disease caused by the use of the accused drug were examined.

Finally, a community study of the above complications was made and the proposed treatment was presented. Also in this study, when examining most of the paradoxical and bimodal complications from the recent studies, an attempt was made to provide a suitable summary in the form of tables for the reader. Also, the study of bimodal side effects along with paradoxical side effects leads to a suitable conclusion regarding the use of TNF‐α inhibitors drugs. Considering the high acceptance of the mentioned drugs by physicians and their high efficacy, it is necessary for physicians especially dermatologists, rheumatologist and gastroenterologist to know the mentioned side effects so that they can apply an appropriate treatment strategy in case of side effects. The results of this study can be a complete summary of the above side effects, which should be practical and attractive to physicians. This is because the above side effects have often been encountered by physicians when treating with this class of drugs, but the lack of knowledge about them has led to the failure of their treatment. The recommended treatment for the above complications depends on the severity of the complication and the type of complication. However, the most common recommended treatment for severe complications is discontinuation of the drug and initiation of specific treatment. In mild cases, it is possible to continue the medication and start treatment of the lesion.

## 2 METHODS

At the beginning of this study and after reviewing the texts and studies, a combination of the following keywords was used:

1. (Anti‐TNF or anti‐TNF or “TNF inhibitor” or adalimumab or infliximab or golimumab or certolizumab) and “disease name” and paradox.
2. (Anti‐TNF or anti‐TNF or “TNF inhibitor” or adalimumab or infliximab or golimumab or certolizumab) and (“side effect” or “adverse effect” or “adverse reaction”).
3. (Anti‐TNF or anti‐TNF or “TNF inhibitor” or adalimumab or infliximab or golimumab or certolizumab) and “disease name”.

Instead of the disease name, the keyword of that disease was inserted, depending on the type of disease.

The above search structure was based on the PubMed database. In other cases, the search was performed keeping the above keywords in the Scopus, Web of Science, and Google Scholar databases. Articles including case reports, case series, observational studies, trials, reviews, and laboratory studies published between January 1, 2000 and June 1, 2022, were searched.

Inclusion criteria:

‐ All relevant articles from PubMed, Scopus, Web of Science, and Google Scholar searches selected based on opinions and reviews of two dermatologists.
‐ Articles must be written in English. If only the abstract of the article was in English, this part was also used when necessary.

Exclusion criteria:

‐ Articles written in a language other than English.

### 2.1 Psoriasis

The incidence of psoriasis and psoriasiform reactions in patients with inflammatory bowel disease treated with TNF‐α inhibitors has been estimated at 6%.^10^ The median duration of occurrence of this adverse effect varied among studies, being 6–12 months in most cases.^11^ However, infliximab was the main cause of these lesions in most studies.^12^, ^13^

Several options are available for the treatment of paradoxical psoriatic lesions. In cases with limited lesions, topical steroids produce an acceptable response in most patients.^14^ Vitamin D analogs or retinoids such as acitretin can be added if needed.^12^ In more severe cases (involvement of more than 10% of the body surface area), several options have been proposed: if the underlying disease is under control, the drug is switched to another TNF‐α inhibitor or discontinued for a period of time. If the underlying disease is not under control, the use of other biologic drugs is suggested. And in both cases, topical steroids, cyclosporine, methotrexate, and phototherapy can be used.^12^

### 2.2 Hidradenitis suppurativa

Only adalimumab is FDA‐approved for the treatment of hidradenitis suppurativa. This disease has been observed after the use of adalimumab, infliximab, and etanercept. Of these drugs, adalimumab plays a greater role than the others.^15^ Specific treatment of hidradenitis suppurativa after treatment with TNF‐α inhibitors is recommended in all cases. In mild disease (Hurley 1), it is recommended to continue treatment with TNF‐α inhibitors. In case of exacerbation or higher degree of disease (Hurley 2,3), it is recommended to discontinue the drug if possible. And if necessary, it is recommended to continue the drug or switch to another TNF‐α inhibitor.^16^

### 2.3 Alopecia

Alopecia after the use of TNF‐α inhibitors includes two types: alopecia areata and psoriatic alopecia.^17^ In the study by Beneh et al., among drug‐induced alopecia, 4.9% of cases were caused by TNF‐α inhibitors. Among TNF‐α inhibitors, the probability of alopecia occurrence was highest after the use of adalimumab. The time interval between the start of drug use and the appearance of lesions averaged 11 months.^17^

In cases of alopecia areata, topical treatment with minoxidil should be given. Discontinuation or continuation of the drug should be evaluated based on benefits and harms.^18^ In rapidly progressive alopecia areata totalis and universalis, switching the drug to ustekinumab may be associated with a good response. However, there are reports of alopecia areata after its use.^17^

### 2.4 Lupus

Because of the increase in TNF‐α in lupus patients and its association with disease severity, especially renal involvement, TNF‐α inhibitors have been proposed for treatment.^19^

Some patients have developed a lupus‐like syndrome after taking TNF‐α inhibitors.^19^ Ten to 10 000 person‐years are affected by this complication, and it accounts for only 0.75% of the complications caused by TNF‐α inhibitors.^20^ The time between the onset of adverse events and initiation of treatment with the drug averages about 10 months.^21^ In a study evaluating 3148 patients with lupus erythematosus from 2000 to 2017, the use of TNF‐α inhibitors was found to increase the incidence of cutaneous lupus by 4.96‐fold and the incidence of systemic lupus erythematosus by 3.64‐fold.^22^

For treatment, it is necessary to discontinue the drug if there is organ involvement. Depending on the type of involvement, topical or systemic corticosteroids along with other immunosuppressive drugs such as methotrexate improve symptoms in most patients.^21^

### 2.5 Dermatomyositis

Several studies have found elevated levels of TNF‐α in serum, muscle, and degenerating tissue in patients with dermatomyositis and polymyositis. This has led to the inhibition of TNF‐α becoming a therapeutic target.^23^ The incidence of dermatomyositis in recipients of these drugs is at the level of case reports and has been reported after the use of all three types of common drugs in this group.^24^, ^25^ The time interval between onset of symptoms and initiation of drug use varied among reports, ranging from 2 months to 4.5 years.^25^, ^26^ In treating patients, discontinuation of TNF‐α inhibitors and use of a systemic corticosteroid alone or together with methotrexate has been associated with favorable response.^26^

## 3 EOSINOPHILIC DERMATOSIS

### 3.1 Eczema

Eczema is one of the most common side effects of TNF‐α inhibitors. The time interval of lesion occurrence from the start of drug treatment ranged from 0.2 to 49.9 months with a mean of 7.1 months.^27^ In a meta‐analysis of 5526 patients with inflammatory bowel disease treated with TNF‐α inhibitors, the incidence of eczema was reported to be 5.5%.^28^ In the treatment of paradoxical eczema, TNF‐α inhibitors do not need to be discontinued in most cases, and treatment can be continued with topical corticosteroids along with calcineurin inhibitors.^29^

### 3.2 Eosinophilia and eosinophilic cellulitis (Wells syndrome)

Wells syndrome includes erythematous plaque lesions that resemble cellulitis and have no specific cause.^30^ Its occurrence after the use of TNF‐α inhibitors, including adalimumab, infliximab, and etanercept, has been reported.^31^, ^32^, ^33^ The time interval between the occurrence of complications and the start of treatment with the drug varied widely, ranging from 2 weeks to more than 9 years.^34^, ^35^ Fifty percent of patients with this syndrome have peripheral eosinophilia.^33^ The first line of treatment for Wells syndrome is low‐dose systemic corticosteroids. Even without treatment, spontaneous improvement occurs within 4–8 weeks. Topical corticosteroids or dapsone may also be used for treatment.^30^

### 3.3 Autoimmune bullous diseases

Autoimmune bullous diseases are nearly common conditions that present with blisters and bullae on the skin and mucous membranes that erode over time.^34^

Several cases of successful treatment of pemphigus vulgaris and pemphigus foliaceus with TNF‐α inhibitors have been reported.^35^, ^36^ Other bullous autoimmune diseases that responded to treatment with TNF‐α inhibitors include cicatricial pemphigus or mucinous pemphigus,^37^, ^38^ IgA pemphigus,^39^ linear bullous IgA dermatosis,^40^ dermatitis herpetiformis,^41^ and epidermolysis bullosa acquisita.^42^ The occurrence of bullous pemphigoid has been reported after treatment with adalimumab, etanercept and infliximab. The time interval between drug use and the occurrence of complications varied from 2 weeks to 3 years.^43^ Regardless of the type of disease, it is recommended to discontinue TNF‐α inhibitors and start specific treatment such as topical or systemic corticosteroids. The addition of other immunosuppressive drugs such as DMARDs may help lesions heal more rapidly.^44^

### 3.4 Lichen planus and lichenoid drug reactions

Severe cases of lichen planus, particularly those with mucosal lesions, have responded to adalimumab, etanercept, and infliximab.^45^

TNF‐α inhibitors have caused lichenoid lesions in a number of patients, termed lichenoid drug eruptions.^46^ These lesions have been reported after the use of adalimumab, infliximab, etanercept, and certolizumab.^46^, ^47^ The time interval between the appearance of the lesion and the start of drug use varied from 3 weeks to 24 months in these patients, although in one case of juvenile rheumatoid arthritis, a lichenoid reaction occurred after 48 months.^48^

In treatment, discontinuation of TNF‐α inhibitors depends on the severity of the lesions and has different results in different cases. However, in most cases, a better response is achieved when the drug is discontinued.^50^ Therefore, it is better to use other drugs in this category or other biologic drugs when possible.^49^ Topical or systemic corticosteroids can be used to treat the lesions, depending on their extent. Other immunosuppressants such as cyclosporine or methotrexate may also be used.^49^

### 3.5 Neutrophilic dermatoses

Neutrophilic dermatoses encompass a wide spectrum of diseases whose common feature is the presence of mature neutrophils in the epidermis, dermis, or hypodermis without evidence of infection.

Several studies demonstrate the efficacy of adalimumab, infliximab, and etanercept in the treatment of pyoderma gangrenosum.^51^, ^52^, ^53^

The paradoxical occurrence of pyoderma gangrenosum has been reported after the use of all TNF‐α inhibitors.^54^, ^55^, ^56^, ^57^, ^58^ Regarding the time interval of lesions, most patients developed symptoms after 3–6 months. However, a period of up to 48 months has also been reported.^54^, ^58^ Three case reports reported paradoxical Sweet syndrome with adalimumab.^59^, ^60^

Discontinuation of TNF‐α inhibitors is the first step in treatment. The next step is specific treatment. In mild cases, local steroids or low‐dose prednisolone may be used. In case of insufficient response or in severe cases, high‐dose prednisolone, methylprednisolone, biologic drugs, or other TNF‐α inhibitors are used.^55^, ^57^

### 3.6 Erythema multiform, Stevens‐Johnson, toxic epidermal necrolysis (TEN)

Of these three conditions, Stevens‐Johnson and toxic epidermal necrolysis have responded well to treatment with infliximab and etanercept. Two systematic review studies indicate that these drugs are effective in the treatment of these two diseases.^61^, ^62^

The paradoxical occurrence of Steven's Johnson and toxic epidermal necrolysis is very rare with these drugs. Only two cases of Steven's Johnson have been reported in studies, both of which occurred after adalimumab use.^63^, ^64^ Although the overall prevalence of erythema multiforme is low, it has been observed more frequently than the above cases. These patients suffered from these lesions after receiving adalimumab, infliximab, and etanercept. These lesions occurred within a few days to 2 weeks after injection.^65^, ^66^

### 3.7 Vasculitis

The effect of TNF‐α inhibitors in the treatment of vasculitis has been reported in several studies. Currently, the use of TNF‐α inhibitors in the treatment of Takayasu arthritis, polyarthritis nodosa, and Behçet's disease has been associated with successful results.^67^, ^68^, ^69^ The time interval between the onset of vasculitis and the initiation of treatment with the drug has varied among studies but has averaged 9.5 months according to the largest study.^70^ Necrotizing vasculitis of medium‐sized vessels has also been reported with a lower prevalence than cutaneous vasculitis of small vessels.^71^, ^72^

No specific treatment method has been proposed for the management of the lesions, but discontinuation of the accused drug and administration of corticosteroids seems to be the best treatment. As for continuing the drug after recovery, other drugs should be used if possible because the relapse rate is high.^70^

### 3.8 Vitiligo

TNF‐α levels are related to skin depigmentation, so TNF‐α levels are higher in affected skin areas than in healthy areas.^73^ In one study, two patients who received etanercept along with UVB irradiation and topical calcineurin inhibitors experienced repigmentation of lesions at the end of the 6th month.^74^

In a cohort study of 11 224 patients, the risk of vitiligo was almost twice as high in the group taking TNF‐α inhibitors as in the control group. Among the drugs in this group, the risk of developing vitiligo was 3.19 for etanercept compared with the control group; it was about 1.7 times higher for infliximab and 1.9 times higher for adalimumab. Of the three drugs mentioned, only etanercept had a statistically significant association with the occurrence of vitiligo.^73^ The occurrence of lesions after certolizomb has also been reported. Considering all TNF‐α inhibitors, the time interval between the appearance of lesions and the use of the drug in a study of 36 patients averaged about 14 months, with a median of 10 months for new cases and 14.5 months for exacerbation of old lesions.^75^

## 4 CONCLUSION

In this study, we sought to examine all true and borderline paradoxical side effects of TNF‐α inhibitors. Although paradoxical side effects are not common in some cases, it is important for physicians to know exactly about these side effects so that if they occur, they can make the best decision for the patient and save him from the physical and psychological consequences. Recognition of the above complications may also be helpful in distinguishing exacerbation of the underlying disease and failure to respond to treatment from the development of a paradoxical complication due to the use of adjuvant medications. The treatment process and management of patients in these two conditions are different, and the proper differentiation of the two will result in physician and patient satisfaction with the treatment process. The treatment approach for the above complications varies depending on the severity of the complication and the type of complication, but the most common treatment approach for severe complications is to stop the drug and start specific treatment. In mild cases, it is possible to continue the medication and start treating the lesion.

## 5 LIMITATIONS AND RECOMMENDATIONS

There are limitations to this study. One of the most important was that not all articles were available. In these cases, only the abstract of the article was used. Also, because the study was a narrative review, no statistical analyzes were performed. For this reason, it is recommended that a meta‐analysis be performed on each part of the study in future studies. Regarding treatment in cases where the therapeutic effect of the drug is not certain, a controlled clinical trial must be conducted to obtain definitive results. In this study, we have discussed more about the classical TNF‐α inhibitors drugs, and the new generation drugs have been discussed less. Therefore, it is suggested that the newer generation of the above drugs be discussed and investigated in future studies.

## CONFLICT OF INTEREST STATEMENT

The authors declare no conflicts of interest.

## Data Availability

All data produced in the present study are available upon reasonable request to the authors.
